# Sexual Size Dimorphism Depends Drastically on Environment: The Case Study in Ground Beetles

**DOI:** 10.3390/insects16121249

**Published:** 2025-12-10

**Authors:** Chiara Ferracini, Enrico Busato, Viktor Alexanov, Igor A. Solodovnikov, Teodora Teofilova, Vladimir Langraf, Alexander Borisovskiy, Sergey Luzyanin, Alexander Ruchin, Dominik Stočes, Anatoliy L. Anciferov, Roman P. Gorbunov, Raisa A. Sukhodolskaya

**Affiliations:** 1 Department of Agriculture, Forest and Food Sciences, University of Turin, 10095 Grugliasco, Italy; chiara.ferracini@unito.it (C.F.); enrico.busato@unito.it (E.B.); 2 State Budgetary Institution of Kaluga Region “Parks Directorate”, 248009 Kaluga, Russia; viktor_alex@list.ru; 3 Department of Zoology, Vitebsk State P.M. Masherov University, 210038 Vitebsk, Belarus; iasolodov@mail.ru; 4 Institute of Biodiversity and Ecosystem Research, Bulgarian Academy of Sciences, 1113 Sofia, Bulgaria; oberon_zoo@abv.bg; 5 Faculty of Natural Sciences, Constantine the Philosopher University, 949 74 Nitra, Slovakia; langrafvladimir@gmail.com; 6 Department of Botany, Zoology and Bioecology, Udmurt State University, 426034 Izhevsk, Russia; borisovscky.alexander@yandex.ru; 7 Department of Ecology and Environmental Science, Kemerovo State University, 650043 Kemerovo, Russia; sl_luzyanin@mail.ru; 8 Joint Directorate of the Mordovia State Nature Reserve and National Park “Smolny”, 430005 Saransk, Russia; ruchin.alexander@gmail.com; 9 Department of Zoology, Fisheries and Hydrobiology, Faculty of AgriSciences, Mendel University in Brno, 613 00 Brno, Czech Republic; xstoces@mendelu.cz; 10 Kostroma Museum-Reserve, 156000 Kostroma, Russia; ancifer.ost@yandex.ru; 11 Belogorye State Reserve, 309342 Borisovka, Russia; xobglor@gmail.com; 12 Research Institute for Ecology and Mineral Wealth Use, Academy of Sciences of the Republic of Tatarstan, 420087 Kazan, Russia; 13 Kazan State Medical University, 420012 Kazan, Russia; 14 Volga-Kama State Biosphere Reserve, 422537 Tatarstan Republic, Russia

**Keywords:** *Carabus granulatus*, sexual size dimorphism, morphometric data

## Abstract

The issue of sexual size dimorphism (SSD) variation is relevant due to the identification of mechanisms by which animals adapt to changing environmental conditions. Using the database of the ground beetle *Carabus granulatus*, compiled from measurements of more than 8000 individuals, we estimated the nature of size variation in beetles captured in different parts of their range, including most of Russia and Europe. ANOVA showed that the sizes of both females and males vary statistically significantly across regions, although not always to the same extent. Therefore, SSD values were assessed not only for each sample by region as a whole, but also for each of the six studied traits (for *C. granulatus* as a whole), as well as the variability of SSD values for each trait in each of the 13 studied populations. SSD was the highest in the southern regions of the species’ range (Bulgaria, Italy) and decreased monotonically northward. The highest SSD values were recorded for elytra length and pronotum width (0.08–0.09), while for the others, they were significantly lower (0.05). Methodological and ideological issues in assessing and applying SSD data in animal populations are discussed.

## Simple Summary

We estimated the nature of size variation in *Carabus granulatus* beetles captured in different parts of their range, including most of Russia and Europe. Our results showed that the sizes of both females and males vary statistically significantly across regions, although not always to the same extent. Sexual size dimorphism (SSD) was the highest in the southern regions of the species’ range (Bulgaria, Italy) and decreased monotonically northward. The highest SSD values were recorded for elytra length and pronotum width. Methodological and ideological issues in assessing and applying SSD data in animal populations are discussed.

## 1. Introduction

Body size (e.g., length or mass) is fundamentally important to animal ecology, behaviour and evolution [1]. For this reason, research on correlations of body size, both in living animals and through deep time, has been a major focus of organismal research [2,3,4]. One commonly studied aspect of body size is sexual size dimorphism (SSD), or body size differences between the sexes. SSD varies in both magnitude and direction (female-biased vs. male-biased) in different clades [5,6]; however, our understanding of the adaptive drivers underpinning the evolution of SSD remains incomplete [7]. Broadly speaking, it is thought that SSD results from a combination of sexual selection, fecundity selection and ecological selection during a species’ evolution [8,9,10]. The intensity of male–male competition (sexual selection) is often thought to modulate selection for large male size; whereas female-biased SSD may arise due to strong fecundity selection where larger females can carry more offspring [8,9,11,12]. However, observed trends may be complicated by the confounding effects of ecology, which can correlate with body size, sexual selection and fecundity selection [10,13,14,15]. On the other hand, body size can differ geographically between particular populations of one animal species, both with latitude and altitude [16,17,18,19] and/or as a result of separation by some geographic barriers [20,21].

This is also shown for ground beetles (Coleoptera: Carabidae), a family of predatory insects with more than 25,000 species distributed worldwide except in polar regions and some oceanic islands. Carabidae is one of the most studied and widespread families of Coleoptera, which specialists around the world have successfully used as a model to demonstrate zoogeographic, phylogenetic, faunistic and ecological patterns [22,23,24,25]. Of particular interest to worldwide research on this well-studied family is the genus *Carabus* Linnaeus, 1758, which includes relatively large and attractive species [26]. In relation to sexual dimorphism in general, in the *Carabus* genus, males of almost all species have ventral surfaces of the three to four tarsal segments of the first pair of legs, thickly covered with pads of setae, which is an adaptation to grasp and hold females during copulation. As for SSD, it is known that females are larger and wider than males [27]. Little is known about morphometric variability within particular species, except for some data concerning sexual dimorphism. The ground beetle, *Carabus granulatus* L., 1758, is widely distributed in Palearctic regions, occurring from the Iberian Peninsula to Eastern Siberia [26]. Based on the wide distribution of *C. granulatus* and its large habitat spectrum, we hypothesized that the species should possibly show morphological variability observed not only in the sexual dimorphism in body size (which is regularly observed in ground beetles) but also in the geographic variability (particular populations are often divided by geographic barriers, especially in mountain massifs and desert areas), which is commonly found in taxa characterized by large geographic ranges.

Thus, the aim of this study was to investigate the variability of size in the ground beetle *C. granulatus*. Specifically, we aimed to (i) investigate the variability of beetle sizes and shapes across geographic gradients; (ii) assess sexual size dimorphism along latitudinal gradient; (iii) evaluate the significance and direction of size changes in *C. granulatus*.

Specifically, we hypothesize that sexual dimorphism in *C. granulatus* is expressed as a larger body size of females and is observed in (most) morphological parameters.

## 2. Materials and Methods

### 2.1. Study Area

**Figure 1:**
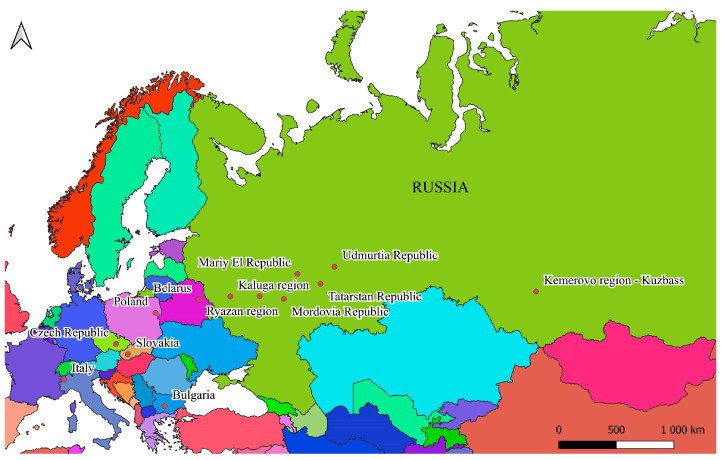
Map of the study area.

Samples were collected from a wide-ranging territory spanning numerous Russian provinces, along with several locations situated in four different European countries (Figure 1).

Specifically, research was conducted at 13 surveyed sites across 7 different countries. All sites with their respective geographical coordinates are reported in Table 1. Beetles were sampled at several points of latitude and longitude: Italy and Kemerovo Province (Russia)—west and east; Bulgaria and Poland—south and north. The other sampling plots were related to the central part of the *C. granulatus* range.

To facilitate this large-scale investigation involving numerous sampled beetles, a high-throughput methodology was developed—an increasingly popular approach in recent decades. This method aggregates information from various scattered publications to synthesize findings on a specific topic [17]. The geographic scope of the sampling was extensive, covering a 17-degree latitudinal and 121-degree longitudinal variance. This latitudinal range was considered sufficient for gradient studies in invertebrate taxa, aligning with previous research [28,29,30]. Within each sampled region, we established at least three collection plots, which differed in parameters such as vegetation type and anthropogenic impact.

### 2.2. Trapping

Fieldwork for beetle collection was carried out in the aforementioned regions throughout the seven-year period of 2018–2024. Morphometric analysis was conducted on the total number of beetles captured within each locality during the defined sampling windows. A consistent experimental design based on Busato et al. [31] was followed, utilizing pitfall traps exclusively. Each trap comprised a plastic receptacle (8.5 cm high; 7.0 cm top diameter; 4.5 cm base diameter) installed flush with the soil surface. A protective lid was employed to prevent the traps from filling with water, reduce evaporation rates, and protect vertebrates. The cups were provisioned with a 6% acidity red vinegar solution—used as bait—and a small amount of dish soap to break surface tension. Field sampling involved deploying ten traps at every study location. Continuous monitoring occurred annually between early April and October. Traps were checked and emptied biweekly (every 15 days) throughout this period. Collected ground beetles were counted, transferred into 50 mL plastic tubes (Sarstedt AG. & Co., Nümbrecht, Germany) containing 70% ethanol for preservation, and subsequently transported to national reference laboratories, where adults were identified to species level using standard dichotomous keys [24]. All specimens belonging to *C. granulatus* were then sexed and arranged in an extended, straightened position on cotton pads. All paddings with captured beetles (or their images) were transferred to Laboratory of Biomonitoring (The Institute of Problems in Ecology and Mineral Wealth, Tatarstan Academy of Sciences) for photographing. All photos were taken by one person using the same method.

### 2.3. Morphometric Data

**Figure 2:**
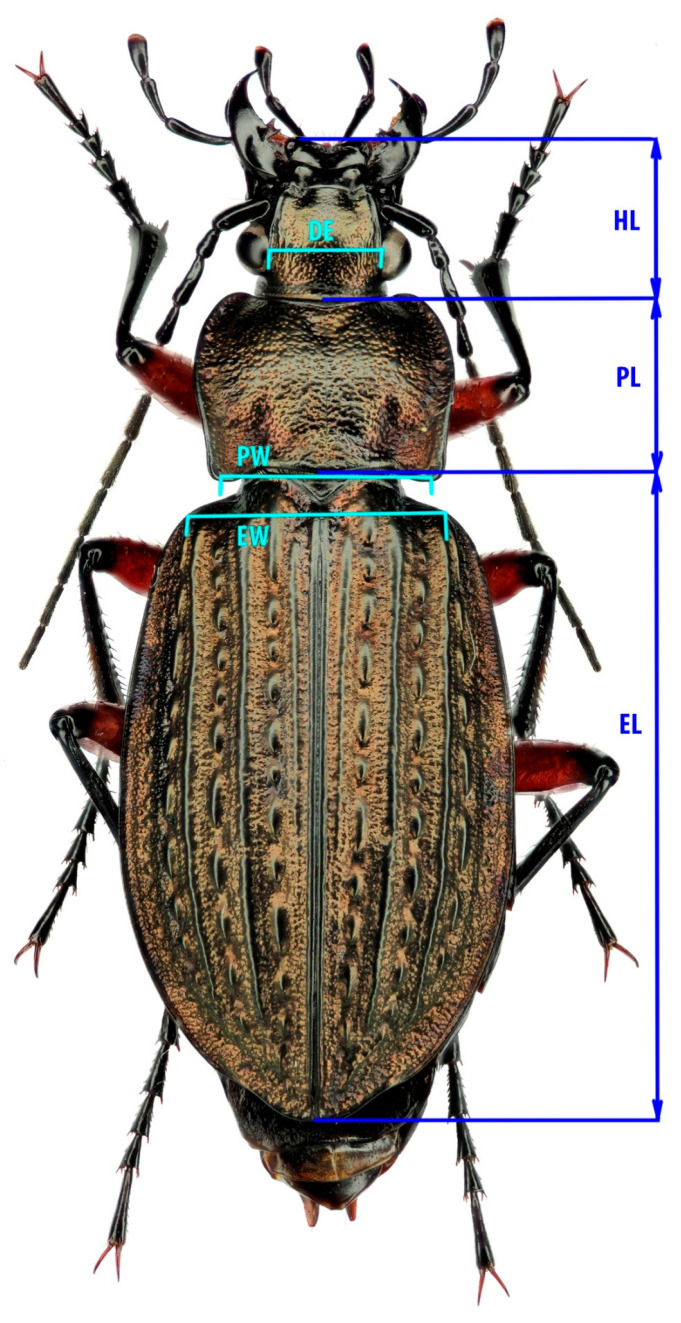
The scheme of measurements in *C. granulatus.* EL—Elytra length: distance between the posterior end of scutellum and the terminus of right elytron (if the right elytron is not intact, the left one may be used). EW—Elytra width: distance between the anterior-distal corners of the elytra. PL—Pronotum length: measured along the central furrow of the pronotum. PW—Pronotum width: distance between the two posterior corners. HL—Head length: distance between the labrum and the juncture of the occiput and postgena. DE—Dorsally measured interocular distance between inner margins. Measured sample size is presented in Table 1.

We selected a range of traits that reflect various aspects of the organism’s biology critical for its successful survival and reproduction (Figure 2). The length and width of the elytra are typically considered proxies for overall beetle size and reflect such traits as fertility, mating success, and survivability (e.g., [32,33,34]). The pronotum is also used as a proxy for body size; moreover, it reflects the beetle’s locomotor performance [35,36], as the muscles of the forelimbs are attached to it. Head length reflects foraging activity, which is important for aspects such as feeding behaviour, mate searching, and orientation in highly fragmented landscapes [37]. The distance between the eyes has been linked to feeding behaviour in relation to light conditions, as many ground beetles hunt at dusk [38].

We developed a high-throughput method for data processing using specialized software designed to measure distances between manually indicated elements in photographic arrays. This software uses a fiducial scale to convert pixel data into real-world measurements; its initial code is publicly available under the MIT license [39]. The images themselves were captured with a Nikon D5100 camera housed within a reflective box setup and featuring a custom opaque light diffuser. The morphometric data utilized for the analysis consisted of six linear scalar float recordings, functioning as dependent variables.

### 2.4. Statistical Analysis

Data analysis was executed in R software (version 4.3.2 [40]). Hypotheses regarding inter-group differences were evaluated using a One-way Analysis of Variance (ANOVA). Prior to this analysis, we conducted prerequisite checks for distributional normality (Shapiro–Wilk test) and homogeneity of variance (Levene’s test). Any violations of these underlying assumptions necessitated the application of suitable corrective data transformations. The main analysis was conducted using the aov() function, which implements the classical ANOVA procedure. The general model structure was as follows:

model <−aov(dependent_variable − independent_variable1 × independent_variable2, data = dataset)

Significance testing of differences between groups was performed using Fisher’s F-test. All statistical tests were considered significant at the *p* < 0.05 level. Data visualization was performed using the ggplot2 package [41].

SSD was assessed according to the methods accepted in global practice [42]:

SSD = (Average value of the trait of females/Average value of the trait of males) − 1

## 3. Results

The size of *C. granulatus* beetles statistically significantly varies across the range in different locations. Table 2 below shows the results for elytra length. For other traits, see Table A1, Table A2, Table A3, Table A4 and Table A5 in Appendix A.

Elytra length showed significant differences across levels of both factors—region and sex—as well as their interaction. In other words, females responded differently to habitat conditions in a given habitat in an area than males.

**Figure 3:**
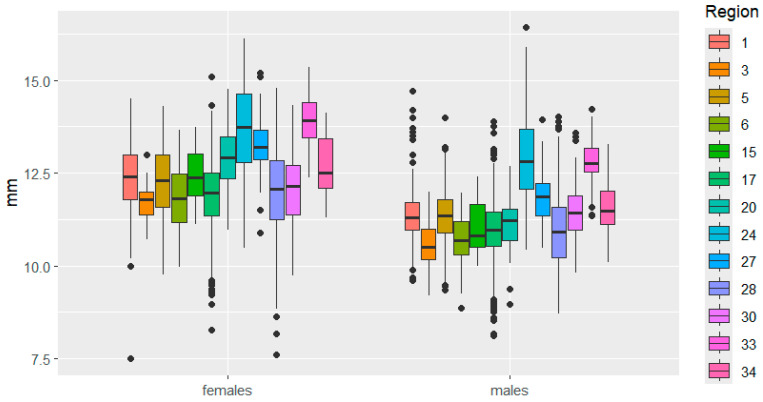
Elytra length variation in *C. granulatus* across different regions. 1—Tatarstan, 3—Kemerovo, 5—Udmurtia, 6—Mari El, 15—Belarus, 17—Kaluga, 20—Bulgaria, 24—Slovakia, 27—Poland, 28—Mordovia, 30—Ryazan, 33—Italy, 34—Czech Republic. Median—the central line within the box representing the 50th percentile of the distribution, dividing the sample into two equal parts; Box—displays the interquartile range (IQR) of the distribution, the lower boundary of the box corresponds to the 25th percentile (Q1), the upper boundary of the box corresponds to the 75th percentile (Q3), IQR is calculated as the difference between Q3 and Q1; Whiskers—represent the range of “normal” values, Calculated as 1.5 × IQR from the box boundaries, can be limited by the minimum/maximum value within this range; Outliers—data points exceeding the whiskers.

**Figure 4:**
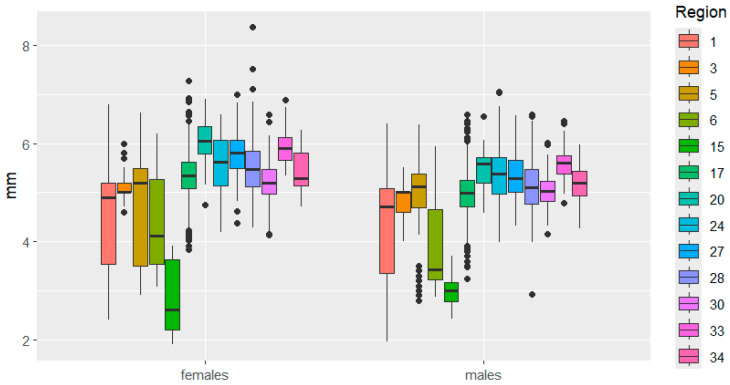
Elytra width variation in *C. granulatus* across different regions. The designations are the same as in Figure 3.

**Figure 5:**
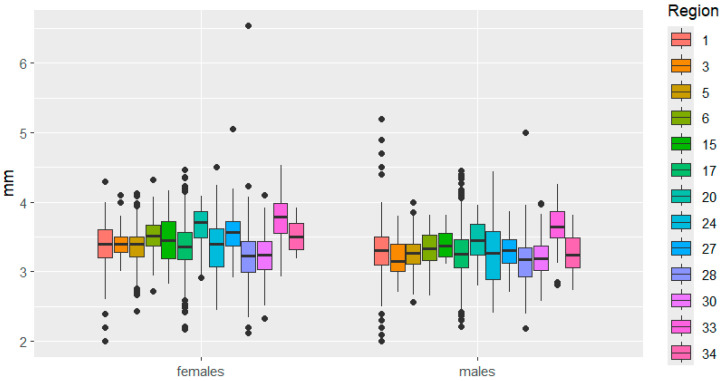
Pronotum length variation in *C. granulatus* across different regions. The designations are the same as in Figure 3.

**Figure 6:**
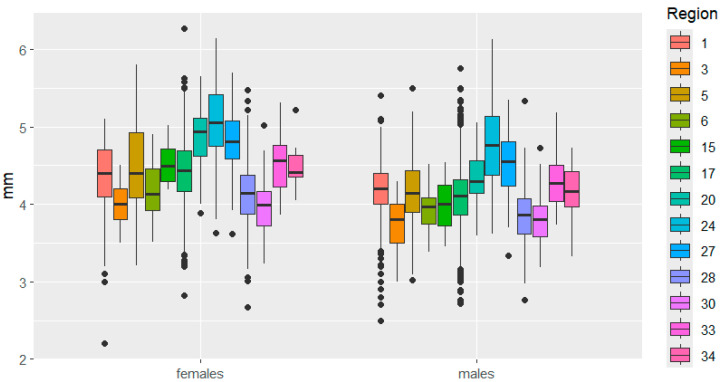
Pronotum width variation in *C. granulates* across different regions. The designations are the same as in Figure 3.

**Figure 7:**
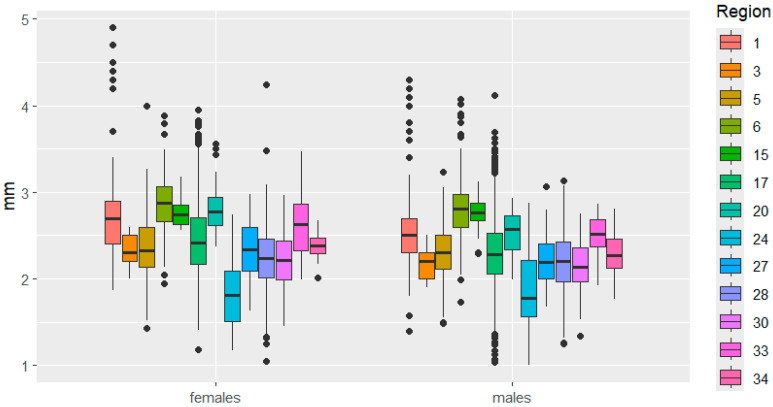
Head length variation in *C. granulatus* across different regions. The designations are the same as in Figure 3.

**Figure 8:**
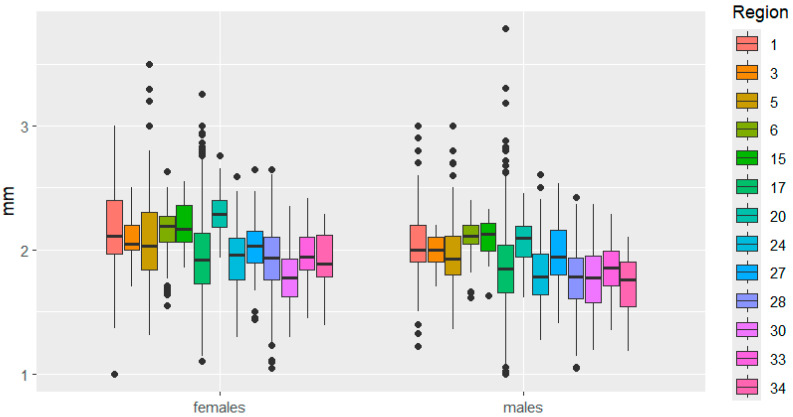
Distance between eyes variation in *C. granulatus* across different regions. The designations are the same as in Figure 3.

This is clearly demonstrated in Figure 3, Figure 4, Figure 5, Figure 6, Figure 7 and Figure 8. It is immediately apparent that females were larger than males in all regions. This indicated that the studied species exhibited a clearly defined SSD. Outliers are most frequent in the Republic of Tatarstan and Kaluga Oblast, which also have the largest samples, making extreme trait sizes more likely to be detected.

Furthermore, significant differences in size variability were observed across regions for individuals of both sexes. For example, elytra length varied significantly between females in Mari El, Belarus, Kaluga, Bulgaria, while it was approximately the same for males. In Mordovia and Ryazan regions, that trait did not differ between females, but it differed significantly between males.

In terms of elytra width, significant differences are observed between the sexes, not so much in the magnitude of the traits, but in the nature of their variability: in the Kemerovo region, it is more pronounced in females, and in the Udmurtia region, in males.

Variability in pronotum size was very similar between the sexes. It is worth noting the small range of variability in pronotum length, while large differences in pronotum width between Belarus and Bulgaria were observed in females.

Head size also varied in some cases in different directions between the sexes. For example, in the Kemerovo—Udmurtia regions, head length is similar in females but significantly different in males. The opposite phenomenon was observed when comparing head length in beetles from the Udmurtia—Mari El regions. In the Mordovia—Ryazan regions, the distance between the eyes varied significantly in females but not in males.

Since ANOVA not only examines the effect of factors on variability but also shows whether the data for each trait differ from the baseline data (in our case, the Tatarstan region as the center of the *C. granulatus* range), we analyzed whether trait sizes in individual regions differ from those of beetles in Tatarstan. The model outputs are presented in Appendix A. Here we present Table 3: regions located at the edge of the studied species’ range, where statistically significant differences from the sizes of beetles living in the center of the range are highlighted in red. The MANOVA analysis presented in Appendix A also showed that beetle sizes differed significantly across regions.

The above differences in variability patterns between females and males required a specific digital illustration. Therefore, we calculated SSD values in three ways. First, for each regional sample, we computed the mean SSD value across the six studied traits. Second, we plotted SSD values for each trait in each of the study regions. Finally, we pooled all samples and calculated SSD values for each trait across the studied geographic range of *C. granulatus*.

**Figure 9:**
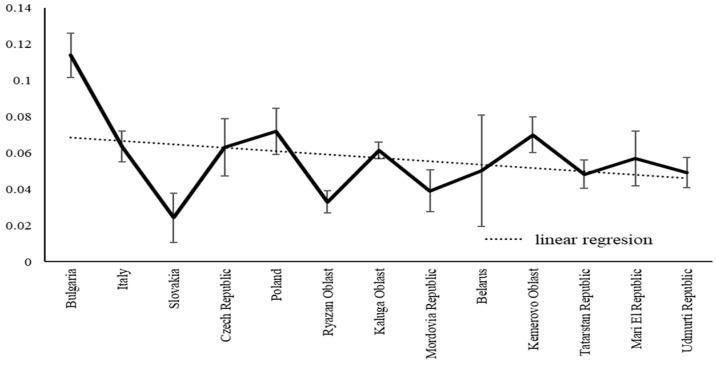
SSD variation in *C. granulatus* along the latitudinal gradient, estimated as the mean of six trait-specific SSD values calculated for each region.

Since male and female variability differed across the studied range points, we further explored this pattern through a series of analyses on SSD variability. Given that latitudinal size variation in *C. granulatus* has already been studied and published, we organized the results by SSD variability along the latitudinal gradient—that is, we assessed how SSD changed for various traits from south to north. In this publication, we focus on presenting specific SSD values, calculated using the well-known Lovich formula (Figure 9). Across the study area, SSD values were consistently positive, indicating that female *C. granulatus* are generally larger than males (Figure 9).

Although the SSD variation curve showed a sawtooth pattern and spikes (due to uneven sampling and habitat representation), SSD decreased monotonically with latitude. The moderate correlation (*r* = 0.44) still reflects meaningful explanatory power, as it aligns with evidence that habitat type affects SSD [43] and beetle life cycles exhibit latitudinal adjustments that lead to changes in body size [44].

**Figure 10:**
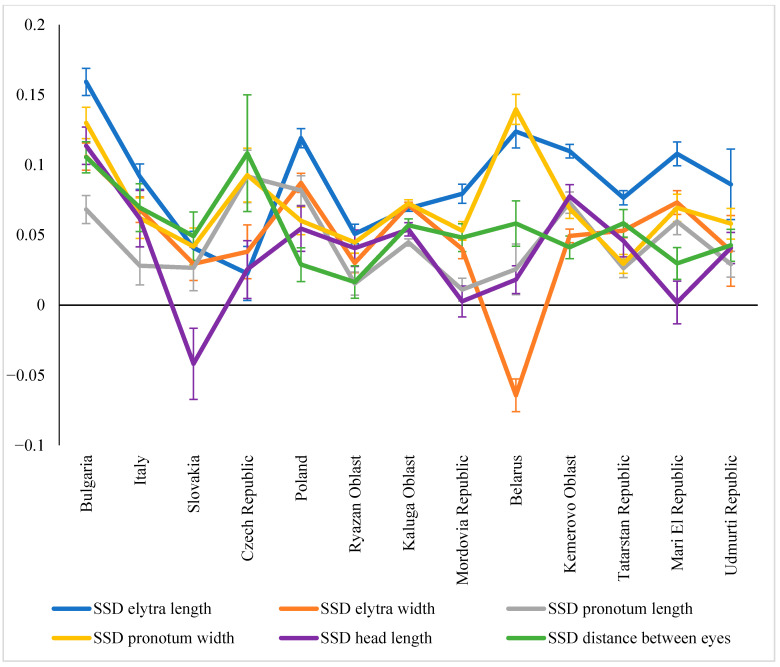
SSD variation for different traits in *C. granulatus* across regions.

When examining SSD variability for individual traits, a mixed picture emerged across the studied regions: SSD values were not equivalent in certain cases (Figure 10). For example, in Bulgaria, significantly higher SSD values were recorded for elytra length, while the lowest were recorded for pronotum length. A similar pattern persisted further north, albeit with a lesser discontinuity. That is, the SSD for elytral length was always higher than for pronotum length.

Cases of inverted SSD, where males were larger than females for a given trait, should also be noted. This has been demonstrated for head length in beetles in Slovakia and elytra width in beetles in Belarus. Head length generally tended to blur the differences between the two sexes, with the SSD for this trait being virtually zero in some regions (Mordovia, Mari El).

**Figure 11:**
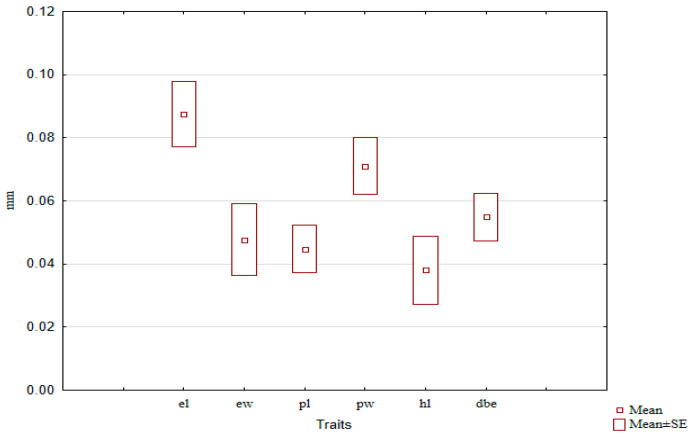
SSD values for different traits in *C. granulatus*, estimated as the mean across the entire sample.

We calculated the SSD values for the entire *C. granulatus* sample and found that the SSD for different traits within the same species exhibited statistically significant differences: the largest SSD was observed for elytra length, and approximately the same for pronotum width. For the remaining linear traits, it was statistically significantly smaller (Figure 11).

## 4. Discussion

Our data confirm previously obtained results on size variability in both *C. granulatus* and other ground beetle species. For example, ten years ago, researchers with a much smaller amount of material at their disposal demonstrated that females of this species are, firstly, larger than males in many respects, and that applying size variability to latitude revealed a monotonic decrease in size towards the north [45]. Those data were later confirmed in another study [46]. The larger size of female ground beetles is a fact confirmed in many carabid species [43,47,48]. The bigger body size found in females is usually explained by the role of this sex in mating behavior. As has been found in many different taxonomic insect groups, females invest much more energy in the reproduction process than males; as a result, a bigger size is much more beneficial for this sex [49]. First, females have to produce eggs, which need to be supplied with substances used in larval development, and second, they also need to find a good place to deposit them. These are among the most crucial conditions in females’ post-copulatory reproductive behavior as they determine the developmental success of their embryos and, as a consequence, have a significant impact on overall reproductive success. In contrast, males usually invest only their sperm; as a result, their energetic costs during courtship are much smaller. All these elements of mating behavior can be easily found in ground beetle species [50].

This clearly suggests that such sexual dimorphism is characteristic of the entire species and does not depend on geographic region. Our research, however, cannot confirm this thesis. According to ANOVA results, the variability of female and male sizes could differ across regions, and, as a result, SSD values would change. It should be emphasized that SSD values varied differently for individual traits. In this case, we certainly agree with the authors of review publications on Bergmann’s rule: if you measure a leg or a head, a female or a male, and conduct research within a continent or on a local scale, the results may be diametrically opposed [51]. Therefore, in our research we tried to apply the basic rules for conducting a large-scale experiment to assess the variability of insect sizes in geographic gradients: We conducted experiments at the intraspecific level, the object was well studied biologically and ecologically (that is, it was known that ground beetles at the imago stage no longer grow), gender was taken into account during measurements, the range of predictor variables was wide, the sample size was in the thousands, and we measured primarily fresh individuals that had not been stored for a long time in fixative liquids. So, we followed the thesis of Shelomi [51], that “Ideal morphometric analyses are intraspecific and within contiguous ranges large enough to include areas of strong changes in biotic or abiotic factors, such as voltinism shifts”.

The traits most closely associated with reproductive fitness (abdomen length in females and genital length in males) are “adaptively canalized.” While this hypothesis is unlikely to explain Rensch’s rule among species or higher clades, it may explain widespread patterns of intraspecific variation in SSD recently documented for many insect species [52].

Fortunately, this remarkable variation in SSD is not without pattern. Rensch (1950) [53] was the first to discern that the magnitude of SSD tends to co-vary with body size. The predominant pattern of covariation, which Rensch characterized as “the rule,” applies to taxa in which males are the larger sex. In such taxa, SSD tends to increase with body size, and hence larger animals are more sexually dimorphic. However, Rensch also noted “by way of exception” that “the opposite correlation applies, i.e., the greater difference is found in the smaller species” in taxa where females are the larger sex.

According to Rensch’s rule, in the case of female-biased SSD, the SSD magnitude in the clade should decrease with increasing size. A comparative analysis of our results with previously published data on other ground beetle species (meaning we analyzed a clade) suggests that this is not the case. For example, in species comparable in size to *C. granulatus*, the SSD averaged across all traits is approximately the same as in *Carabus odoratus* [47] or lower in *Carabus adamsi* [46]. In addition, in another, larger ground beetle species, *Carabus exaratus*, the SSD values are indeed lower (according to Rensch’s rule) [44]. However, in ground beetle species with a body size much smaller than the species we studied, SSD values are not higher (as required by Rensch’s rule), but lower [48]. The figures presented here refer to average SSD values calculated for several traits.

The main result of our study is that SSD can vary when analyzing several traits.

Therefore, the Rensch’s rule manifestation varies depending on the studied trait. In insect SSD studies, 1–2 traits are typically used. Among the dimensional traits, these are body length (water striders, dragonflies, caddisflies), thorax length (diopsids, fruit flies, sepsids, scatopsids), and hind tibia length (water striders, dung flies). In ground beetles, only elytral length is usually included in the SSD analysis. Without diminishing the importance of this trait for carabids, we emphasize that the variability of elytral size may differ from that of other parts of the insect body; therefore, depending on the trait selected, SSD values can vary significantly. This is also noted by other researchers [54,55].

Our results show that larger SSD values belong to elytra length and pronotum width. In most cases, SSD of traits changes unidirectionally along the latitudinal gradient; however, in some regions, certain traits demonstrate multidirectional sexual dimorphism. In some instances, individual traits may display inverted sexual dimorphism, with higher values in males. Consequently, studying the variability of sexual dimorphism based on a single trait may yield contradictory results when attempting to interpret general patterns beyond that specific trait. To avoid this ambiguity, we employed a simple integrative approach—calculating the mean value of SSD across all studied traits. The results indicate a trend towards a decrease in sexual size dimorphism along the latitudinal gradient from south to north. This trend may be associated not only with direct functional adaptations to local climatic conditions but also with changes in the life cycle. However, testing these hypotheses requires separate investigations.

Previous studies on the size variability of *C. granulatus* along the latitudinal gradient revealed a tendency for body size to decrease towards the north [46]. This finding partially contradicts the so-called temperature–size rule, which states that ectotherms in warmer conditions grow faster as juveniles, mature at smaller sizes, and reach smaller maximum body sizes [56]. However, some studies suggest that this rule may represent a special case of a trade-off between size, fecundity, and fitness, governed by more nuanced patterns [57,58].

Thus, our results suggest that sexual dimorphism increases with increasing body size in *C. granulatus*. Sexual dimorphism may increase via two scenarios: either females become disproportionately larger, or males become disproportionately smaller. Our findings indicate that both sexes increase in size when moving southward; however, females exhibit greater changes in body size relative to males, which leads to the observed increase in sexual dimorphism. Therefore, the more pronounced directional response of females to environmental changes produces this effect.

These findings are directly related to the challenge of confirming Rensch’s rule, which posits that male-biased SSD generally increases with average body size (in species where males are the larger sex). Most studies on this topic have focused on mammals and birds, where Rensch’s rule is often observed. Yet, even in birds, the rule is not observed in species where females are the larger sex, leading some Authors to conclude that Rensch’s rule does not exist as an independent scaling phenomenon [6]. In insects, Rensch’s rule is observed in only half of the species studied and, apparently, is not a universal rule for this class. Moreover, it is worth noting that both the number and quality of studies (specifically, the presence or absence of phylogenetic correction) vary significantly across orders. For example, in Diptera and Coleoptera, SSD and its dynamics have been assessed in several families. In contrast, data for other orders are often combined across all families, or only a single family within the order has been examined (e.g., caddisflies). On the other hand, the data confirming Rensch’s rule in Diptera and Coleoptera are quite scattered. Among these, studies on ground beetles (Carabidae) are extremely rare and typically examine SSD for only one trait. And different environmental sensitivities between sexes in certain species can also lead to results that do not align with the rule, likely due to variations in environmental factors, population cycles, and selection pressures, which can cause contradictory results, as well.

## 5. Conclusions

The differences in trait variation between the sexes of the ground beetle *C. granulatus* demonstrated in the article lead to the conclusion that SSD is not a constant value in the animal kingdom. Its value can vary both across the studied population as a whole and among different linear traits within the same ecological context. This raises questions about the methodology for assessing SSD and the design of studies, which should be conducted using uniform measurements and include large samples of the studied species. In this case, it is preferable to conduct research at the intraspecific level to eliminate phylogenetic noise.

## Figures and Tables

**Table 1:** Regions of sampling plots, geographical coordinates, number of plots, and sample size.

№	Region	Region Number	Latitude	Longitude	Number of Plots	Sample Size
1	Bulgaria	20	42.8° N	22.9° E	1	94
2	Italy	33	45.4° N	10.3° E	2	105
3	Slovakia	24	47.8° N	17.6° E	3	143
4	Czech Republic	34	50.0° N	32.4° E	2	66
5	Poland	28	52.7° N	22.9° E	4	160
6	Ryazan Oblast ^a^	30	53.9° N	44.2° E	3	271
7	Kaluga Oblast ^a^	16	54° N	36° E	8	4528
8	Mordovia Republic ^a^	28	54.7° N	44.4° E	13	456
9	Belarus	15	55.2° N	30.2° E	1	132
10	Kemerovo Oblast ^a^	3	55.3° N	86.1° E	3	208
11	Tatarstan Republic ^a^	1	55.7° N	49.1° E	12	2418
12	Mari El Republic ^a^	6	56.6° N	47.1° E	2	215
13	Udmurtia Republic ^a^	5	56.85° N	53.20° E	1	142

Legend: the apex a corresponds to Russia. The region number refers to a database of ground beetles that is constantly updated as beetles are collected from new regions. The species *C. granulatus*, analyzed in our study, was not collected in all regions of the database. Therefore, the region numbering is not sequential.

**Table 2:** Two-way ANOVA results when analyzing elytra length variation in *C. granulatus*.

	Df	SumSq	MeanSq	F Value	Pr (>F)
Region	13	1612	124	196.89	<2 × 10^−16^
Sex	1	1671	1671.1	2653.39	<2 × 10^−16^
Region:sex	13	44	3.4	5.39	8.68 × 10^−10^
Residuals	8227	5181	0.6		

**Table 3:** Impact of factors “Region” and “Region × Sex” on trait size variation in *C. granulatus*. Asterisks indicate statistically significant differences across regions relative to the reference area (the Republic of Tatarstan, approximated as the center of the range for *C. granulatus*). Red highlighting denotes regions at the periphery of the global sample—those farthest from the range core.

Trait/Region	3	5	6	15	17	20	24	27	28	30	33	34
EL	*		*		*	*	*	*	*	*	*	*
EW	*	*		*	*	*	*	*	*	*	*	*
PL	*		*			*		*	*	*	*	
PW	*	*	*	*	*	*	*	*	*	*	*	
HL	*	*	*		*	*	*	*	*	*		*
DBE	*				*	*	*	*	*	*	*	*
Trait/Region×Sex												
EL				*		*		*		*		
EW		*	*	*	*	*		*	*			
PL	*					*		*				
PW		*		*	*	*			*			
HL							*		*			
DBE										*		

## Data Availability

The original contributions presented in this study are included in the article. Further inquiries can be directed to the corresponding author.

## Appendix A

**Table A1:** Two-way ANOVA results when analysing elytra width variation in *C. granulatus*.

	Df	SumSq	MeanSq	F Value	Pr (>F)
region	13	1449.5	111.5	330.444	<2 × 10^−16^
Sex	1	156.3	156.31	463.229	<2 × 10^−16^
region:sex	13	38.2	2.94	8.707	<2 × 10^−16^
Residuals	8227	2776.1	0.34		

**Table A2:** Two-way ANOVA results when analysing pronotum length variation in *C. granulatus*.

	Df	SumSq	MeanSq	F Value	Pr (>F)
region	13	40.3	3.1	31.101	<2 × 10^−16^ ***
Sex	1	23.7	23.71	237.818	<2 × 10^−16^ ***
region:sex	13	3.3	0.25	2.509	0.00198 **
Residuals	8227	820.1	0.1		

Statistical significance was defined as *p* < 0.05. The *p*-values are represented as follows: ** = *p* < 0.01, *** = *p* < 0.001.

**Table A3:** Two-way ANOVA results when analysing pronotum width variation in *C. granulatus*.

	Df	SumSq	MeanSq	F Value	Pr (>F)
region	13	217	16.69	112.21	<2 × 10^−16^ ***
sex	1	161.5	161.5	1085.8	<2 × 10^−16^ ***
region:sex	13	10	0.77	5.17	2.82 × 10^−9^ ***
Residuals	8227	1223.6	0.15		

Statistical significance was defined as *p* < 0.05. The p-values are represented as follows: *** = *p* < 0.001.

**Table A4:** Two-way ANOVA results when analysing head length variation in *C. granulatus*.

	Df	SumSq	MeanSq	F Value	Pr (>F)
region	13	184.3	14.18	106.766	<2 × 10^−16^ ***
sex	1	26.5	26.512	199.615	<2 × 10^−16^ ***
region:sex	13	4.1	0.317	2.383	0.00343 **
Residuals	8227	1092.7	0.133		

Statistical significance was defined as *p* < 0.05. The *p*-values are represented as follows: ** = *p* < 0.01, *** = *p* < 0.001.

**Table A5:** Two-way ANOVA results when analysing the distance between eyes variation in *C. granulatus*.

	Df	SumSq	MeanSq	F Value	Pr (>F)
region	13	75.3	5.796	70.171	<2 × 10^−16^
Sex	1	16.8	16.798	203.375	<2 × 10^−16^
region:sex	13	1.7	0.129	1.563	0.0879
Residuals	8227	679.5	0.083		
